# Investigation of Genetic Alterations in Congenital Heart Diseases in Prenatal Period

**DOI:** 10.1055/s-0041-1736566

**Published:** 2021-11-09

**Authors:** Emine Ikbal Atli, Engin Atli, Sinem Yalcintepe, Selma Demir, Rasime Kalkan, Cisem Akurut, Yasemin Ozen, Hakan Gurkan

**Affiliations:** 1 Department of Medical Genetics, Faculty of Medicine, Trakya University, Edirne, Turkey; 2 Department of Medical Genetics, Faculty of Medicine, Near East University, Nicosia, Cyprus

**Keywords:** CHD, aCGH, genetic testing, congenital heart disease

## Abstract

The prenatal diagnosis of congenital heart disease (CHD) is important because of mortality risk. The onset of CHD varies, and depending on the malformation type, the risk of aneuploidy is changed. To identify possible genetic alterations in CHD, G-banding, chromosomal microarray or if needed DNA mutation analysis and direct sequence analysis should be planned.

In present study, to identify genetic alterations, cell culture, karyotype analysis, and single nucleotide polymorphism, array analyses were conducted on a total 950 samples. Interventional prenatal genetic examination was performed on 23 (2, 4%, 23/950) fetal CHD cases. Chromosomal abnormalities were detected in 5 out of 23 cases (21, 7%). Detected chromosomal abnormalities were 10q23.2 deletion, trisomy 18, 8p22.3-p23.2 deletion, 8q21.3-q24.3 duplication, 11q24.2q24.5 (9 Mb) deletion, and 8p22p12 (16.8 Mb) deletion. Our study highlights the importance of genetic testing in CHD.

## Introduction

Congenital anomalies are the important factor for infant death and congenital heart disease (CHD) that account for 30 to 50% of these deaths. 1 Therefore, prenatal diagnosis and management of fetal cardiac abnormalities are important because they provide information before birth and gives chance to decision of treatment options before and after delivery and plan for specific health essentials at birth. There is a few information which shows interaction between prenatal diagnosis and morbidity or mortality of affected fetuses. 2 Screening of fetal cardiac anomalies has been applied between 18 and 22 weeks of gestation. 3

The onset of fetal arrhythmias, myocarditis/cardiomyopathy, heart failure, valvular insufficiency or obstruction, and cardiac tumors varies. But on the other side, prenatal detection of small ventricular or atrial septal defects, minor valve lesions, partial anomalous pulmonary venous connection, and coronary artery anomalies is not possible. According to the study, sensitivity and specificity of first trimester ultrasound examination for detection of major CHD were 85 (95% confidence interval [CI]: 78–90) and 99% (95% CI: 98–100), respectively. 4

Fetal echocardiography has been suggested according to the American Heart Association, the American Society of Echocardiography, and the Pediatric and Congenital Electrophysiology Society guidelines. 3 To be able to identify genetic abnormalities, fetal genetic assessment has been indicated in fetuses with cardiac defects. 5 6 7 8 9 10 In general, abnormal karyotype is detected in affected cases. 11 Depending on the malformation type, the risk of aneuploidy varies. 3 Aneuploidy risk was 46 to 73% in atrioventricular septal defect, 19 to 78% in truncus arteriosus, 6 to 43% in double-outlet right ventricle/conotruncal malformations, 5 to 37% in coarctation/arch interruption, 4 to 16% in tricuspid valve dysplasia, 7 to 39% in tetralogy of Fallot, 0% in heterotaxy/cardiosplenic syndromes, and 0% in transposition of great arteries. The most known genetic alteration is 22q11 deletion that was associated with several cardiac anomalies, including interrupted aortic arch, truncus arteriosus, ventricular septal defect, and tetralogy of Fallot. 12 Genetic abnormalities can be identified by using G-banding or chromosomal microarray. 13 14 If these tests are normal and there is a positive family history of a similar cardiac defect or long QT syndrome and Noonan syndrome, DNA mutation analysis and direct sequence analysis should be planned to identify genetic alterations ( Table 1 ). Advancement of technology, next-generation-based techniques like whole exome sequencing will be used to identify new genetic syndromes in fetuses suspected with CHD. 15 16

**Table 1:** CHDs and associated genetic syndromes

Genetic abnormality	Genetic test	Type of CHD	% Affected by CHD	Associated genes
Trisomy 21	FISH CMA	ASD, VSD, AVCD, TOF	40–50	3rd copy of chromosome 21; unbalanced translocation
Trisomy 13	FISH CMA	ASD, PDA, VSD, pulmonary atresia with CHD	60–80	3rd copy of chromosome 13; unbalanced translocation
Trisomy 18	FISH CMA	ASD, VSD, PDA, CoA, bicuspid aortic valve, complex CHD	60–80	3rd copy of chromosome 18
Monosomy X (Turner syndrome)	FISH CMA	CoA, BAV, AS, partial anomalous pulmonary, venous return, HLHS	23–50	Partial or incomplete absence of 1 X chromosome 45X
DiGeorge syndrome (chromosome 22q11.2 deletion)	FISH, CMA	IAA type B, aortic arch anomalies, truncus, arteriosus, TOF	70–75	22q11.2 deletion
Noonan syndrome	Noonan gene panel	PS, hypertrophic CM, ASD	70–80	RAS-MAPK pathway (KRAS, SOS1, RAF1, NRAS, BRAF, SCHOC2, CBL, RIT1)
Alagille syndrome	CMA	PS, TOF	90	JAG1, NOTCH2
Holt-Oram syndrome	Molecular testing	HCM	75	TBX5

Abbreviations: AVCD, atrioventricular canal defect; ASD, atrial septal defect; BAV, bicuspid aortic valve; CHD, congenital heart disease; CM, cardiomyopathy; CMA, chromosomal microarray; CoA, coarctation of the aorta; HCM, hypertrophic cardiomyopathy; PDA, patent ductus arteriosus; PS, pulmonic stenosis; TOF, tetralogy of Fallot; VSD, ventricular septal defect.

## Methods

A total 950 samples were collected prospectively between the years of 2014 to 2020. The study population was referred for prenatal diagnosis for the suspicion of CHD risk. This study was approved by the Ethics Committee of the Faculty of Medicine, Trakya University, and all methods were performed in accordance with the relevant guidelines and regulations. Written informed consent was obtained from the parents of the CHD fetus. Results of fetal heart screening, maternal and gestational age, and extracardiac abnormalities were recorded. Evaluation of pregnancy was performed by Perinatology Unit, Department of Obstetrics, and Trakya University Faculty of Medicine. Ultrasound examination was performed by physicians with prenatal ultrasound diagnostic qualifications using GE Voluson E8 (GE Healthcare, Lafayette, Colorado, United States), Philips iU22 (Philips, Copenhagen, Denmark), Siemens Acuson Sequoia 512 (SIEMENS, Munich, Germany), or S2000 (SIEMENS, Munich, Germany) color Doppler ultrasound with 2D/3D volume probe (frequency: 4, 0–8, 0 MHz). All exams included a two-dimensional evaluation of cardiac structures with the “basic” (four-chamber view of the fetal heart) and the “extended basic” cardiac screening examination (views of the outflow tracts). Ductal and aortic arches position was evaluated by color Doppler. Cardiac situs, rhythm, venous inflow, atrial and ventricular chambers, atrioventricular and semilunar valves, and ventriculoarterial connections were assessed. 17 18 According to our protocol, the results were classified as “normal” or “abnormal.” The cases considered “abnormal” were undergone to a postnatal echocardiogram at the same hospital. We categorized the abnormal fetal heart according to complexity of the heart anatomical abnormalities in “complex,” “significant,” “minor,” and “others.” 19 20 Twenty-three pregnant women underwent fetal ultrasound examination and fetal heart screening at the mid-pregnancy stage.

## Fetal CHD Genetic Examination

Amniotic fluid (20–30 mL) was drawn by Department of Obstetrics for cell culture, karyotype analysis, and single nucleotide polymorphism-array analysis. The specimens were sent to the laboratory for cell culture, and after that G-banding and karyotype analysis have been done by using a chromosome automatic analyzer (US PE Company). The karyotype was nomenclatured according to the International Cytogenetics International Nomenclature System (AKAS Karyotyper Genetic Diagnosis Assisted Karyotyping System, supported by Istanbul Technical University, Ministry of Science and Technology, Istanbul, Turkey). The statistical analyses were performed using SPSS Statistics version 19.0 (SPSS Inc, Chicago, Illinois, United States), and 2-tailed *p* < 0.05 was considered statistically significant.

## Results

A total 23 (2, 4%) out of 950 pregnancies were diagnosed as a fetal CHD by systemic ultrasound and fetal echocardiography ( Table 2 ). The mean age of the pregnant women was 28.3 years (range: 22–41) and the median gestational age at the time of ultrasound examination was 21.2 (range: 18–23).

**Table 2:** Indications for fetal echocardiography of 23 fetuses with cardiac defects

Indication	*n* (%)
Suspicion of cardiac anomaly in a routine scan	12 (56.52)
Family history	3 (13.04)
Extracardiac defect	3 (13.04)
Complex	5 (21.73)

The prenatal findings are summarized in Table 3 . Interventional prenatal genetic examination was performed for 23 (2, 4%, 23/950) fetal CHD cases. Among these, chromosomal abnormalities were detected in 5 out of 23 cases (21, 7%).

**Table 3:** The prenatal findings of 23 fetal CHD cases

Patient age	Type of defect	Karyotype/aCGH results
25	Bilateral choroid plexus cyst, hyperechogenic focus in the heart on US	10q23.2 deletion
41	Cystic hygroma, VSD in the heart, hyperechogenic bowel	Trisomy 18
22	Left heart hypoplasia, VSD, cystic hygroma, posterior fossa anomaly	8p22.3-p23.2 deletion, 8q21.3-q24.3 duplication
24	Dextrocardia	11q24.2q24.5 (9 Mb) deletion
28	Hyperechogenic focus in the heart	8p22p12 (16.8 Mb) deletion

Abbreviations: aCGH, array comparative genomic hybridization; CHD, congenital heart disease; US, ultrasound; VSD, ventricular septal defect.

A total 22 (95.65%) out of 23 fetuses with isolated cardiac malformations were delivered and alive at a mean follow-up of 38 (range: 1–120) months. Among the infants with isolated cardiac lesions delivered alive, conotruncal anomalies and univentricular lesions were the predominant types of defects. All fetuses with a prenatal diagnosis of isolated severe dysrhythmias are currently alive.

## Discussion

Accuracy of antenatal diagnosis and the clinical impact of the antenatal recognition determines the capability of identification of fetal cardiac defects. Many cardiac anomalies are severe and couples do not continue to the pregnancy. But in our study population, the vast majority of our patients decided to continue their pregnancy after the diagnosis of fetal CHD. During the decision stage in general, the severity of the anomaly had an influence on the decision. In our study, there was no difference in the decision to terminate the pregnancy with an isolated anomaly compared with cases with multiple anomalies (20, 3 vs. 24, 7%). Whether the prenatal diagnosis of a cardiac malformation is beneficial for the affected infants has been the subject of many discussions. 21 The incidence of prenatal CHD varies from 2, 4 to 54%. 5 22 23 24 25 26 This variability was based on the performance of a systematic screening in each country. The number of CHD patients was lower than other study groups in the literature, and this was a limitation of our study. Statistical analyzes of our study population were made accordingly, due to the low number of abnormal cases. Because the number of abnormal cases are low and this was prevented, a more detailed statistical analyses of our study population. However, our chromosomal anomality rates were higher and also we have a higher incidence of complex cases. Here, we recommended that continuous efforts should be needed for prenatal screening programs of CHD. We believe that with the knowledge of these data, we can improve the outcomes of morbidity and mortality of children in our institution.

Patient 1 was 18 weeks pregnant and had a bilateral choroid plexus cysts and cardiac hyperechogenic focal findings on ultrasound examination. After array comparative genomic hybridization (aCGH) analyses, deletion of 10q23.2 region was detected. According to the literature, CHDs were associated with recurrent 10q22q23 deletion syndrome and partially overlapping distal 10q23.2.q23.31 microdeletions. 27

*BMPR1A* gene plays a key role during the development of CHD in patients suffering from interstitial 10q deletions. Previous studies showed that *BMPR1A* was the highest ranked candidate gene for CHD. Cardiac specific deletion of BMPR1A (alk3) disrupts the cardiac morphogenesis in mice and causes the ventricular septum, trabeculation, and endocardial cushion. 28

*ALK3* was also important during the morphogenesis of atrioventricular valves and annulus fibrosus and studies demonstrated that inactivation of Alk3 in the atrioventricular canal myocardium causes abnormalities. 29 This cardiac phenotype was consistent with our patients who carried a larger 10q deletions (atrioventricular septal defect [AVSD], atrial septal defect, VSD, and tricuspid insufficiency).

In a case who underwent amino synthesis at 18 weeks of gestation because of left heart hypoplasia, VSD, cystic hygroma, posterior fossa anomaly, we detected 8p22.3–p23.2 deletion and 8q21.3–q24.3 duplication. Babies who carry 8p23 deletions do not develop any problem during pregnancy and delivered via normal birth. After delivery, they can be identified. In our study population, structural anomalies were detected as a result of ultrasound examination. According to the literature, heart defects developed in 75% of children with a terminal deletion of 8p23 and 94% of those with an interstitial deletion. The *GATA4* gene (GATA binding protein 4) was located proximal part of band 8p23.1 and responsible for heart conditions. 30 Spectrum of heart defects varies. AVSD accounts for almost half of the heart conditions in children who carry an 8p23 deletion. Pulmonary stenosis, atrial septal defects, ventricular septal defects, hypoplastic left heart syndrome, and congenital diaphragmatic hernia were also reported during the 8p23 deletion. Newborn babies who have CHD may have respiratory distress and may require oxygen and/or breathing assistance. Surgery is necessary to repair the hole. According to the Wat studies interstitial 8p23 deletion and terminal 8p23 deletion were detected in affected cases. 31 But on the other side there were limited literature information regarding the 8q duplication. In one series, 30 babies out of 33 were born with a heart defect. In another series, nine babies out of 20 were born with a duplication from 8q13–24 to 8qter had a heart defect. The typical heart defect in babies with 8q duplications is known as a conotruncal defect. Recombinant eight syndrome occurs in as many as two-thirds of babies. Conotruncal defects arise 5 to 6 weeks after conception when the heart is still an S-shaped tube with upper and lower bulges. 32 33

In one patient, a right-located heart anomaly was reported in our study population. We detected 11q24.2q24.5 (9 Mb) deletion and this result was confirmed by aCGH analysis. When we compared the phenotype of our patient with literature that describes a new patient carrying the large interstitial 11q deletion who exhibits the Jacobsen syndrome (JBS; OMIM 147791) phenotype. To our knowledge, only five patients with an overlapping deletion in 11q24.3q25 have been reported. 34 35 36 37 38 Thrombocytopenia, developmental delay, CHD, and short stature were the characteristic clinical findings of 11q deletion. 39 The breakpoints are located to the distal to subband 11q23.3 and the deletion usually extends to the telomere and interstitial deletions rarely occurred within the JBS region. In our patient, 11q24.2q24.3 deleted region contains eight OMIM genes: ETS1, FLI1, SENCR, KCNJ1, KCNJ5, ARHGAP32 (RICS), TP53AIP1, and BARX2. ETS1 and FLI-1 were the members of the ETS family of transcription factors and ETS-1 plays an important role in a wide range of biological functions. Recent studies showed that ETS plays key role during the heart development in nonmammalian and mammalian species. 40 41 42 43 44 These findings indicated that ETS-1 should be a candidate gene for CHD in our patient. 35

## Conclusion

Prenatal diagnosis is important in CHD. After ultrasonic examination, genetic evaluation of pregnancy should be planned. Depending on the malformation type, the risk of aneuploidy varies. To identify possible genetic alterations, G-banding, chromosomal microarray or if needed DNA mutation analysis and direct sequence analysis should be planned. Our study highlights the importance of genetic testing in CHD.
